# *Sarcoptes* Infestation. What Is Already Known, and What Is New about Scabies at the Beginning of the Third Decade of the 21st Century?

**DOI:** 10.3390/pathogens10070868

**Published:** 2021-07-09

**Authors:** Katarzyna Talaga-Ćwiertnia

**Affiliations:** Jagiellonian University Medical College, Faculty of Medicine, Chair of Microbiology, Department of Infection Control and Mycology, 31-008 Kraków, Poland; katarzyna.talaga@uj.edu.pl; Tel.: +48-12-633-0877 (ext. 231)

**Keywords:** *Sarcoptes* infestation, ordinary scabies, crusted scabies, scabies prevalence, scabies treatment, scabicide

## Abstract

Currently, there are three known subtypes of scabies: ordinary, crusted, and bullous. The worldwide prevalence of scabies remains high in the 21st century. To decrease the social, economic, and psychological impact on the enormous population infected, a lot of important work has been completed over the last 20 years concerning the management of scabies. For example, a standardization of guidelines for the treatment of scabies has been completed and programs have been designed for the prevention and treatment in endemic populations, called mass drug administrations. Unfortunately, these only apply to the ordinary form of scabies. Moreover, resistance to the drugs currently used in treatment is growing, which imposes the need to search for new treatments. For this purpose, new acaricides are being developed to enhance the therapeutic options for the patients’ benefit and effectively treat this disease. There is also the necessity for prevention before the development of scabies. An effective vaccine has the potential to protect people before this disease, especially in endemic areas. Unfortunately, there are no such vaccines against *Sarcoptes* yet.

## 1. Introduction

Scabies is a skin infestation caused by *Sarcoptes scabiei* var. *hominis* [1,2,3]. It was recognized as a disease in ancient India, China, and the Middle East [4]. In 1687, Bonomo and Cestoni described *Sarcoptes* mites as a cause of scabies, but the first author who accurately described the parasite was De Geer in 1778 [5,6]. The disease was quite common during the Napoleonic wars and the American Civil War. Pandemics of scabies occurred between 1919–1925, 1936–1946, and 1964–1979 during military conflicts [7]. Crusted scabies was described for the first time in Norwegian leprosy patients in 1848 [8]. Bullous scabies was described for the first time by Bean in 1974 [9]. In the 19th and 20th centuries, research into scabies was not a priority, mainly as *Sarcoptes* infestation was considered a disease that generally affected poor people. During World War II, Kenneth Mellanby et al. provided studies on mite biology, transmission, and treatment options [10,11]. The main advancement of research on the pathophysiology and host–parasite interactions of scabies was carried out at the turn of the 21st century, mainly through experimental animal models [12,13]. It is known that scabies causes a significant social effect, lowers the quality of life, and impacts an enormous population economically and psychologically. Nevertheless, for years, scabies was absent from the global health agenda [14]. To improve diagnostic methods, effectively treat patients infected with scabies, and search for new drugs, a global advocacy body, the International Alliance for the Control of Scabies (IACS), was formed in 2012. In 2017, scabies was included by the WHO in the group of Neglected Tropical Diseases, which are influenced by IACS, and many studies that document the morbidities and burden caused by the diseases have been completed since then [15,16]. Today, the most important issue, and challenge, is to devise effective strategies for the population-level control of scabies [17].

## 2. Biology and Pathophysiology of *Sarcoptes* Infestation

Scabies is a highly contagious ectoparasitic skin infestation. It is caused by *Sarcoptes scabiei* var. *hominis* [18], but also with other *S. scabiei* variants, such as *S. scabiei* var. *canis* or *S. scabiei* var. *suis.* These variants are normally responsible for pseudo-scabies, which are self-limiting and considered non-transmissible from human to human [19]. *S. scabiei* var. *canis* can adapt themselves to a human substrate, in rare cases, causing outbreaks of scabies in immunosuppressed patients [20].

Scabies can come in several forms, but the most common form is ordinary scabies, also called classical, typical, standard, usual, or normal scabies [1,17]. In the case of a primary infestation, individuals are usually asymptomatic for the incubation period of 4–6 weeks, but with subsequent infestations, *Sarcoptes* infection symptoms develop much more rapidly, from hours to days. An itch and skin lesions, most commonly small, scattered papules, often with excoriation and in some cases burrows, are symptoms of ordinary scabies [17]. 

The second form is known as crusted scabies (sometimes called Norwegian scabies, but this name should no longer be used) [1,21]. This kind of scabies is more severe than ordinary scabies and is more common in immunosuppressed patients. The immune system is overwhelmed and unable to defend against the mites on the skin, resulting in mite hyperinfestation of the host skin [22]. Crusted scabies could also occur also in patients affected by neurological diseases that cause reduced sensation, patients with a reduced ability to scratch due to immobility, and in genetically susceptible patients [23].

The other subtype of scabies, which seems to be the most severe, is bullous scabies (also called bullous pemphigoid-like eruptions) with atypical symptoms mimicking bullous pemphigoid [24,25] but with a negative Nikolsky’s sign [26]. In bullous scabies, the mites or their secretions may cause alterations or the release of bullous pemphigoid antigen, leading to an immunological response and resulting in the production of antibasement membrane zone antibodies, as seen in bullous pemphigoid [27]. Another explanation is that the mite itself may act as an antigen that cross-reacts with the bullous pemphigoid antigen to stimulate the production of autoantibodies [28].

Transmission of scabies occurs predominantly through skin-to-skin contact with an already infected individual, including through sexual contact. As scabies is contagious, the other way of getting infected is via contact with infected clothing, towels, or bedding, which is rare for common scabies but may occur with crusted scabies [16,17,21]. Admittedly, adult *S. scabiei* die outside their human host within 24–36 h, but immature mites can survive for one week, contributing to the spread of scabies [29]. Carriage of scabies is especially common during the first weeks (a 4- to 6-week period) when scabies infestation is usually asymptomatic. After this time, pruritus (itching) occurs, which is the effect of sensitization to mite antigens [3].

The direct effect of scabies is itching, mediated by nonhistaminergic itch mechanisms, which leads to scratching. However, it is not an obligatory symptom of *Sarcoptes* infestation [3,17]. The itch may vary in severity, from an extremely severe reaction affecting the quality of life (disturbing patients’ sleep, interfering with concentration at work or school) to minor complaints [17,30,31]. It may be localized to the site of visible scabies lesions or generalized to other body parts [17]. The reduced or completely absent itch sensation is characteristic for patients affected by neurological diseases that cause reduced sensation [23].

Pruritus leads to breaches in the skin barrier that create an entry point for *Staphylococcus aureus* and *Streptococcus pyogenes* and is the reason why scabies can lead to bacterial infection of the skin (impetigo) [32,33]. The associated complications of *Sarcoptes* infections can be divided into skin and soft tissue infections, e.g., cellulitis, skin abscess, necrotizing fasciitis, and septicemia; immune-mediated diseases, such as renal diseases; and even rheumatic heart disease [14,34,35]. It has been proven that the parasites themselves directly impact the occurrence of associated bacterial infections by modulating the microenvironment around the mite and reducing the innate immunity of the infected human. This is accomplished by mite gut proteins, such as serpins and serine proteases, that impact, among others, the host’s complement defense and neutrophil function [36,37,38,39,40]. The association between *Sarcoptes* and pathogenic bacteria is observed mainly in tropical or subtropical regions [14]. Mason et al. and Romani et al. suggest that, especially among young children, a high percentage of impetigo lesions can be linked to scabies (up to 40%) [41,42]. Epidemics of acute glomerulonephritis in Trinidad or Southern Africa may be explain by the earlier occurrence of scabies epidemics in these areas. The reduction in hematuria in children following scabies treatment or a reduction in impetigo or skin sore prevalence in parallel with a reduction in scabies numbers may be explained by the association of scabies infection with its complications [43,44,45,46,47]. Complications associated with *Sarcoptes* infestation may even lead to death of infected people [6,13,16,23,48].

## 3. Clinical Features of *Sarcoptes* Infestation 

In classic scabies, the papules are small, often excoriated with hemorrhagic crusts on top. The pathognomonic sign of ordinary scabies are burrows, which appear as thin, brown-grey lines of 0.5–1 cm. The detection of burrows may be difficult due to excoriation or secondary bacterial infection. Other lesions that may be observed in the course of classic scabies are vesicles (usually at the start of a burrow), nodules (firm, 0.5 cm in diameter), and weals [21]. Typical scabies lesions stem from a hypersensitivity response to mite products and are also possibly caused by temporary excavations of immature mites [17].

Skin lesions in crusted scabies consist of generalized, erythematous, fissured plaques covered by scales and crusts. The plaques have a yellow-to-brown, thick, verrucous aspect when localized on bony prominences (e.g., finger articulations, elbows, and iliac crest). Crusted scabies may also occur as diffuse non-crusted scabies with the involvement of the back [21]. 

Bullous scabies are characterized by a variable inflammatory infiltrate, predominantly neutrophils or eosinophilic spongiosis or both at the same time, with a subepidermal split. An intraepidermal blister has also been reported. These pathological features are similar to bullous pemphigoid. Occasionally, in this form of scabies, mites or eggs can be found in the bullae [49].

In the scabies subpopulations, lesions are found frequently in some body areas and rarely in others (Table 1). Multiple body surfaces are involved in many cases, but the distributions may differ in infants, children under 2 years old, and adults. Nevertheless, the regions containing lesions are roughly symmetrical across the left and right sides of the body [17].

## 4. Epidemiology of *Sarcoptes*

Risk factors for scabies include young age, presence of many children in the household, low family income, poor housing, sharing clothes and towels, and irregular showering. Elderly age also predisposes people to being infected by *Sarcoptes*. Nevertheless, any age group can be involved. Both sexes are equally affected, as are all races [6,52]. Healthy individuals (with normal immune responses) can be infected by ordinary scabies [21]. Crusted scabies infections most frequently occur in immunocompromised patients, those with HIV infection, acute myeloid leukemia (AML), lepromatous leprosy, kidney transplantation, diabetes, Down’s syndrome or other intellectual disabilities, dementia, neuromotor disorders, or those using immunosuppressants [3,6,53]. As with crusted scabies, bullous scabies can occur in immunosuppressed individuals [54,55]. Bullous scabies affects mainly males and especially individuals older than 70 years [49].

Sometimes clinicians have difficulty making the right diagnosis due to multiple morphologies of scabies, and the variation of clinical subtypes (Table 1, Table 2). In 2020, the International Alliance for the Control of Scabies (IACS) Criteria were proposed that should be used for the diagnosis of classic scabies. The 2020 IACS Criteria are not intended for use in the diagnosis of variant or atypical presentations of scabies, such as crusted scabies, bullous scabies, scabies in immunocompromised individuals, or scabies in the elderly, cognitively impaired, or bedridden individuals [17].

Previously administered treatments, including systemic or corticosteroid ointments, often modify the symptoms and signs and may cause inappropriate diagnosis [17]. Classic scabies misdiagnosis and inappropriate treatment with steroids may exacerbate *Sarcoptes* infestation symptoms and cause progress to crusted scabies (Table 2).

The reason for improper diagnosis may be caused by a failure to recognize atypical localizations of scabies, for example, toenail infestations [60,61].

## 5. Laboratory-Based Diagnosis of Scabies 

The major goal of scabies diagnostics is confirming the diagnosis by accurate and rapid identification of the parasite. In this context, the diagnosis of scabies is still challenging and potentially time consuming. While different laboratory-based techniques for scabies have been developed in the last few decades, only a few could be used as universal tools in areas of high endemicity and in countries of low endemicity [62].

**Figure 1:**
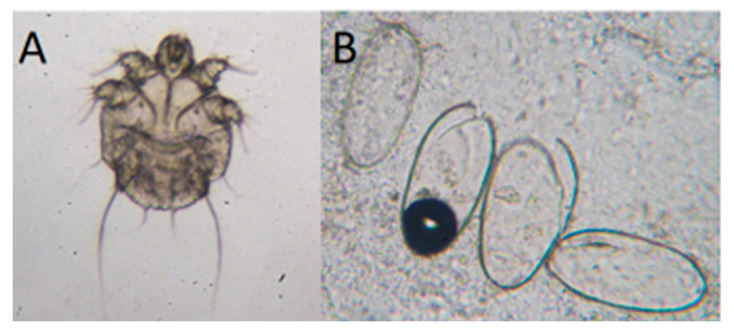
Optical microscopy examination of skin scrapings for the diagnosis of *Sarcoptes scabiei* as revealed by mites (**A**) and eggs (**B**) in light microscopy. Magnification 400×.

A rapid and reliable method to diagnose scabies (level A in the guidelines by IACS 2020) is to visualize mites, eggs, or fecal pellets through optical (light) microscopy (4–400×) of material taken from skin lesions (Figure 1) [62]. It seems that staining with Calcofluor White may also be a good diagnostic tool in visualizing *Sarcoptes* [61], but more studies are needed.

A negative microscopic test does not exclude the diagnosis, as microscopy is frequently negative in patients with clinically diagnosed scabies. As with many other laboratory tests, the reliability of light microscopy is highly operator dependent [17,62].

It seems that the direct examination of indoor dust may be useful in identifying the presence of *S. scabiei* in the indoor environment and can be used as an additional tool in the diagnosis of scabies [20].

High powered imaging (video dermoscopy, low-cost videomicroscopy, and reflectance confocal microscopy with at least 70–1000× and 30–400× magnification, respectively) and dermoscopy allow for detailed non-invasive visualization of scabies mites in vivo and can confirm the diagnosis of scabies [17]. 

More recently, serological testing and different molecular techniques have been developed as diagnostic methods for scabies. Several serological assays for *Sarcoptes* have been developed, and detection of targeted scabies antibodies using immunoassays has shown promise for future use. Nevertheless, the antigens targeted by these tests require more trials to eliminate cross reactions (including house dust mites such as *Dermatophagoides*) and to enhance the sensitivity, specificity, and reproducibility of the tests [62]. Molecular identification methods, such as PCR amplification and its modifications, seem to be the most accurate and rapid way for diagnosing scabies, with potential use in countries of low endemicity, but the utility of these methods requires more study [62,63,64]. 

Generally, a major drawback is that the guidelines for diagnostic tests are prepared only for ordinary scabies and do not exist for crusted or bullous scabies [17].

## 6. Scabies Prevalence

Scabies is one of the most common dermatological disorders present in all parts of the world, with a large number of new cases especially in developing countries. The incidence of scabies varies depending on patients’ country of residence and age, being endemic in a number of countries in tropical and subtropical regions. The highest prevalence of scabies reported in the general population is in Asia, Oceania, and Latin America [16,65]. In low- and middle-income countries and tropical regions, scabies disproportionately affects children [3]. The highest rates of scabies in children occur in Central America, the Pacific Islands, and Northern Australia [6]. In contrast, in the developed world, scabies generally causes outbreaks in health institutions and vulnerable communities [6]. The Global Burden of Disease study estimated that in 2015–2017, over 145,000–200,000 people in the world were infected with *Sarcoptes scabiei* [65,66,67]. In 2016, the direct effects of scabies infestation on the skin alone were estimated to have resulted in nearly 3.7 million disability-adjusted life years (DALY, years of life lost due to premature mortality plus years lived with disability) [68]. Moreover, in high prevalence areas, scabies is a major underlying cause of bacterial skin infections and serious complications affecting cardiovascular and renal function [6]. 

## 7. Outbreaks

Many researchers emphasize that delayed or misdiagnosed *Sarcoptes* infection may lead to serious consequences, such as extensive outbreaks of the infestation, for example, in health institutions and among nursing home residents and personnel [16,69,70,71,72]. In high-income countries, scabies outbreaks also occur in homeless populations and groups living in crowded conditions such as schools, prisons, and child-care facilities [16]. Poor living conditions and overcrowded refugee shelters also provide an ideal environment for the spread of scabies and increase the risk of outbreaks [3,16]. Outbreaks have also been observed in natural disaster victims following drought, flooding, and earthquakes. A long-term analysis (1984–2013) by Mounsey et al. demonstrated that crusted scabies is the most common infection outbreak in institutions compared to the other forms [71]. Outbreaks caused by *Sarcoptes* also remain an important problem in the army and during military conflicts; for example, in 2013, there was an outbreak in the British Army [6]. 

## 8. Treatment of Scabies

**Figure 2:**
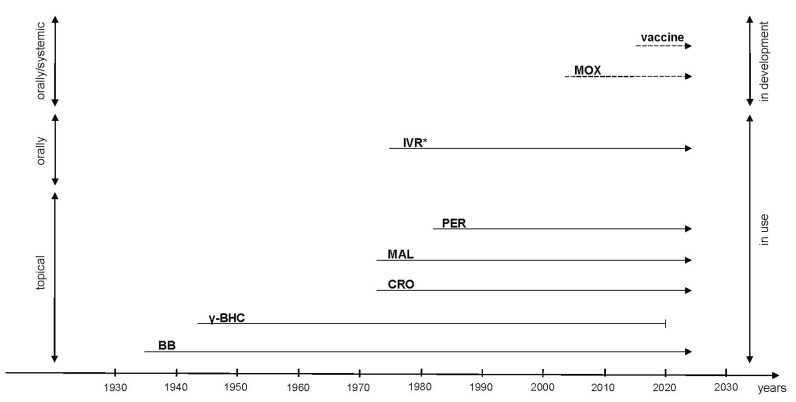
Timeline of treatment options for scabies—(author’s own elaboration based on [21,73]). * The Nobel Prize in Physiology or Medicine in 2015 for the discovery of ivermectin, BB—benzyl benzoate, γ-BHC—benzene hexachloride, CRO—crotamiton, MAL—malathion, PER—permethrin, IVR—ivermectin, MOX—moxidectin, - - - - - - - -> during tests, -------------> currently used, -----|withdrawn from use.

One of the most important scabicides currently used to treat scabies is ivermectin, which was discovered during the 1970s/1980s (Figure 2). It considerably increased the therapeutic options for the management of *Sarcoptes* infestation [16]. Nevertheless, the current treatment options for scabies possess important limitations. The treatments are sometimes ineffective in preventing relapse and inflammatory skin reactions. Treatment options are not always safe, especially in children and pregnant women. Furthermore, there has been an emergence of resistance among scabies mites to the standard acaricides [17].

In general, for individual patients, a number of treatment options are currently available including a topical scabicide. In practice, the treatment options are limited and differ due to the availability of scabicides in each country, due to medicine regulations and other issues. In consequence, usually, patients do not have access to a variety of treatment options. Topical preparations should be applied at night and left in place for 8–12 h [6,21,74,75]. A second application is recommended after 7–14 days. All the patient’s close personal contacts should be treated simultaneously to avoid infestation. European principles of scabies treatment from 2017 indicate that topical treatment should be applied to all skin regions including the skin beneath the ends of the nails [21].

In the treatment of bullous scabies, topical gamma benzene hexachloride, sulfur ointment/cream, malathion, benzyl benzoate, permethrin or orally ivermectin, or topical ointment combined with ivermectin orally have been used [49].

To cure nail scabies, application of scabicide ointment under occlusive dressing is needed. Moreover, treatment may include additional procedures, such as frequent nail trimming, applications of urea cream or other topical keratolytics, including 5% salicylic acid, and mechanical brushing of the nails (in water) for removal of the mites [58,60,76,77,78]. Removal of dystrophic nails was also described as necessary to a successful outcome [77,78].

After treatment of scabies, a common side effect is an itch, which may persist for up to 2–4 weeks. Post-treatment itch should be treated with repeated application of emollients. In some cases, oral antihistamines and mild topical corticosteroids may also be used [21].

To assess the effectiveness of treatment, a microscopic examination is recommended two weeks after completion of scabicides [21].

To achieve success in treatment, not only is ordering the proper scabicide needed, but washing of clothing, bedding, towels, and other items is needed; these items should be machine washed (at least at 50 °C), dry-cleaned, or sealed and stored in a plastic bag for 1 week [21].

## 9. Mass Drug Administration (MDA)

Mass population treatment (mass drug administration, MDA) is recommended for control in populations that are predisposed to outbreaks, for example, in endemic countries, mass population displacements, and in the management of epidemics in closed communities. In the MDA model of treatment, all individuals should be treated irrespective of symptoms and the choice of treatment is oral ivermectin because it is easier to administer than topical scabicides [21]. MDA strategy seems to be efficient, especially in communities with a scabies prevalence higher than 5% [16].

In recent years, scabies MDA and other schemes for neglected tropical diseases eradication programs (such as onchocerciasis or trachoma) have been tested. The combining of MDA options was found to be effective and safe in the first trials [16].

## 10. Difficulties with Treatment

Treating children has been challenging because, for many years, the treatment of scabies in children was not well defined and may not have led to a complete therapeutic effect [70,79]. Moreover, it was not clear if and what side effects might appear after treatment with scabicides. Due to this, treatment was selected according to the age, the extent of the changes that may affect the whole body in children, and the availability of the drugs. Currently, the guidelines define the specific applicability of the appropriate acaricides and the doses that can be used in infants and older children. Permethrin is licensed for use in children from age 2 months onwards. Ivermectin should not be used in children weighing less than 15 kg [21]. The treatment of pregnant women was also controversial for many years due to poor data on the side effects of the available acaricides. The newest treatment principles recommend permethrin, benzyl benzoate, and sulfur as safe treatment options in pregnancy [21].

Side effects of the currently used scabicides that are reported by patients include itching experienced for several days after treatment, which significantly reduces the comfort of life [17].

Treatment failure with regard to *Sarcoptes* has been documented in the literature and may be due to inadequate drug administration, duration of treatment, incorrect dose, or drug resistance. For example, Aussy et al. reported that topical benzyl benzoate alone and a single dose of ivermectin (vs. two intakes) were the cause of treatment failure in a 112-patient multicenter study [2]. On the other hand, Sunderkötter et al. explained that permethrin treatment failure may be due to inadequate exposure to this acaricide [1,80]. Furthermore, Isogai et al. explained that *Sarcoptes* mites and eggs can survive in nails and subungual debris [81]. Therefore, it is important to evaluate the typical and atypical locations of scabies-induced lesions each time a diagnosis is made so as not to overlook rarely affected areas (e.g., toenails) that cause recurrence of *Sarcoptes* and the need for re-treatment [61,79,81] and to follow the guidelines as regards the use of appropriate doses of drugs and exposure time. Relapses in untreated, or even after treatment of, cutaneous lesions of crusted or ungual scabies may lead to several episodes of crusted scabies per year [60,82]. Moreover, the patient’s general condition (e.g., immunosuppression) may influence therapy by prolonging it or requiring the use of several acaricides [83]. It is probable that mass treatment programs may also have an impact on drug resistance to scabicides and the treatment failure, including therapy with permethrin and ivermectin [21].

## 11. New Treatment Options of Scabies

Due to the need to use new scabicides with better activity against eggs and with half-lives long enough to cover the whole 14-day life cycle of the mite, new trials are underway. Dose-ranging experimental studies to determine whether higher doses of ivermectin are more effective in controlling scabies infestation were tested [84]. 

Moxidectin, as a replacement of ivermectin in oral administration, is also being tested. Moxidectin belongs to the same family as ivermectin [85]. Importantly, it is a drug with rapid absorption, large distribution, and a much longer half-life in plasma and the skin than that of ivermectin [52,85]. Due to this pharmacological characteristic, it may potentially cover the entire lifecycle of the scabies mite [52,86]. In a pilot trial in the experimental pig model for scabies, a single dose of moxidectin used orally was found to be more effective than the conventional 2 doses of ivermectin at a one-week interval [85]. There is a multicenter clinical phase II trial in humans in progress [84].

Additionally, there are novel therapies in development and testing, including herbal compounds [87,88] and entomopathogenic fungi [89]. Medical plants are safe, effective, and patient-friendly natural treatment methods for scabies. Essential oils and plant extracts may be ordered in different forms: as a shampoo (extract of *Azadirachta indica*), paste (*Curcuma longa*, *Aegle marmelos,* Cayenne), syrup (*Solanum nigrum* L, *Tinospora cordifolia*) or added to a hot water bath (*Capsicum annuum*) [90]. The use of plant-based products raises high hopes, but there are several problems that will surely affect the introduction of these preparations in the treatment of patients infected with *Sarcoptes*. One of them is the possibility of causing contact dermatitis. Medical plant agents require careful studies on humans, as the current knowledge confirms their effectiveness in vitro or in animals [90]. Some of these agents are limited by geographical locations. Moreover, the composition of oils cannot be patented, as it differs depending on the place of occurrence of the plants the oils are made from [52].

It seems that *Sarcoptes* gut proteases, such as serine protease, aspartic protease, and cysteine protease, have a synergistic effect when given along with permethrin and ivermectin. This may be a way to avoid mechanisms of resistance during drug metabolism and to improve the efficacy [52].

Although *S. scabiei* are largely species-specific, *S. scabiei* var. *canis* can survive and occasionally establish in non-canine hosts as well. For example, as mentioned earlier, it could be responsible for outbreaks in immunocompromised patients [20] and in many mammal species. In recent years, the importance of pets as “family members” has been growing systematically. In this context, studies have proven the effectiveness of orally and topically applied fluralaner treatment. Fluralaner is a novel treatment option for canine sarcoptic mange, which can be administered as a single dose and is safe and effective and results are maintained for twelve weeks after treatment [91].

## 12. Vaccines

Vaccines are expected to be a solution to prevent the development of scabies, especially in endemic areas and for crusted scabies [82]. The interactions between *Sarcoptes* and the infected person’s immune system, which have been better understood in recent years, should positively influence research into the production of a vaccine. An anti-mite vaccine has been tested in an animal model, in rabbits [92,93] and mice [94], but more trials are needed.

## 13. Summary

The worldwide prevalence of scabies remains high in the 21st century. To decrease the social, economic, and psychological impact on the enormous population infected, a lot of important work has been completed over the last 20 years concerning the management of scabies. Many actions have been taken, including the standardization of guidelines for the treatment of scabies. Mass drug administration is a program designed and used for prevention and treatment in endemic areas. Unfortunately, resistance to the drugs currently used in the treatment of scabies is growing, which imposes the need to search for new treatments. For this purpose, new acaricides are being developed to enhance the therapeutic options for the patients’ benefit and effectively treat this disease. There is also the necessity for prevention before developing scabies, especially in people living in endemic areas. Unfortunately, studies to create an effective vaccine have not yet been successful.

## Figures and Tables

**Table 1:** Differentiation of scabies subtypes and their localizations in adults, children, and infants (author’s own elaboration based on [17,26,49,50,51]).

Form of Scabies	Localization			
	Typical		Atypical	
	Adults and Children	Infants	Adults and Children	Infants
Ordinary	hands, fingers, web spaces, wrists, elbows, axillae, umbilicus, belt line, nipples, buttock, penis shaft, genitals in men, areolae in women	torsos, palms, soles, wrists	head, scalp, neck, palms, soles, nails	nails
Crusted	palms, fingers, feet, hands, soles	generalized	head, earlobes, toenails	toenails
Bullous	arms, trunk, genitals, groin, legs, chest, back, feet, buttocks, thighs, neck, wrists, generalized	generalized with predominance on trunk and hands, fingers, wrists	head	ND

ND—not described.

**Table 2:** Differentiation of scabies from other skin diseases (author’s own elaboration based on [21,56,57,58,59]).

Subtypes of Scabies	Other Skin Diseases
Ordinary	arthropod bites, folliculitis, impetigo, papular urticaria, atopic dermatitis, contact dermatitis, nummular eczema, prurigo nodularis, bullous pemphigoid (urticarial stage), dermatitis herpetiformis, lice infestation, delusional parasitosis, Morgellons disease
Crusted	hyperkeratotic eczema, dyshidrotic eczema, psoriasis, Darier’s disease, palmoplantar keratoderma, contact dermatitis, pityriasis rubra pilaris, seborrheic dermatitis, atopic dermatitis, erythrodermic mycosis fungoides, Sézary syndrome
Bullous	bullous pemphigoid, dermatitis herpetiformis, Darier’s disease Letterer–Siwe disease, lupus erythematosus, bullous arthropod bites, bullous impetigo, pemphigus vulgaris, incontinentia pigmenti (inflammatory stage)
